# EGFR- and Integrin α_V_β_3_-Targeting Peptides as Potential Radiometal-Labeled Radiopharmaceuticals for Cancer Theranostics

**DOI:** 10.3390/ijms25158553

**Published:** 2024-08-05

**Authors:** Cibele Rodrigues Toledo, Ahmed A. Tantawy, Leonardo Lima Fuscaldi, Luciana Malavolta, Carolina de Aguiar Ferreira

**Affiliations:** 1 The Institute for Quantitative Health Science & Engineering, Michigan State University, East Lansing, MI 48824, USA; toledoci@msu.edu (C.R.T.); tantawya@msu.edu (A.A.T.); 2 Comparative Medicine and Integrative Biology, College of Veterinary Medicine, Michigan State University, East Lansing, MI 48824, USA; 3 Department of Physiological Sciences, Santa Casa de Sao Paulo School of Medical Sciences, São Paulo 01221-020, Brazil; leonardo.fuscaldi@hotmail.com (L.L.F.); luciana.malavolta@gmail.com (L.M.); 4 Department of Radiology, Michigan State University, East Lansing, MI 48824, USA; 5 Department of Pharmacology & Toxicology, Michigan State University, East Lansing, MI 48824, USA; 6 Department of Biomedical Engineering, Michigan State University, East Lansing, MI 48824, USA

**Keywords:** EGFR, integrin α_V_β_3_, cancer, theranostics

## Abstract

The burgeoning field of cancer theranostics has witnessed advancements through the development of targeted molecular agents, particularly peptides. These agents exploit the overexpression or mutations of specific receptors, such as the Epidermal Growth Factor receptor (EGFR) and α_V_β_3_ integrin, which are pivotal in tumor growth, angiogenesis, and metastasis. Despite the extensive research into and promising outcomes associated with antibody-based therapies, peptides offer a compelling alternative due to their smaller size, ease of modification, and rapid bioavailability, factors which potentially enhance tumor penetration and reduce systemic toxicity. However, the application of peptides in clinical settings has challenges. Their lower binding affinity and rapid clearance from the bloodstream compared to antibodies often limit their therapeutic efficacy and diagnostic accuracy. This overview sets the stage for a comprehensive review of the current research landscape as it relates to EGFR- and integrin α_V_β_3_-targeting peptides. We aim to delve into their synthesis, radiolabeling techniques, and preclinical and clinical evaluations, highlighting their potential and limitations in cancer theranostics. This review not only synthesizes the extant literature to outline the advancements in peptide-based agents targeting EGFR and integrin α_V_β_3_ but also identifies critical gaps that could inform future research directions. By addressing these gaps, we contribute to the broader discourse on enhancing the diagnostic precision and therapeutic outcomes of cancer treatments.

## 1. Introduction

The area of cancer theranostics has seen remarkable progress, with the development of several targeting agents, particularly peptides. These molecules exploit the upregulation of specific receptors, such as the Epidermal Growth Factor receptor (EGFR) and integrin α_V_β_3_. These receptors recognize specific amino acid sequences like tyrosine kinase domain-targeting peptides and RGD (Arg-Gly-Asp) derivatives, respectively, making them prime targets for therapeutic and diagnostic applications [1,2]. 

Peptides are particularly notable in this context due to their low molecular weight, generally consisting of up to 50 amino acid residues. This attribute allows for enhanced penetration into tumor tissues and swift clearance from the bloodstream and non-targeted tissues, which improves imaging accuracy and reduces treatment-related toxicity. Additionally, peptides are characterized by low antigenicity, and they can undergo chemical modifications during the radiolabeling process without losing activity, further enhancing their suitability for theranostic applications [3,4]. Over the past decades, the identification and utilization of peptides that target EGFR and integrin α_V_β_3_ have significantly increased.

In this comprehensive review, we provide a thorough summary of the current literature concerning the utilization of radiolabeled EGFR- and integrin α_V_β_3_-targeting peptides in cancer imaging and peptide receptor radionuclide therapy (PRRT). We discuss the various EGFR- and integrin α_V_β_3_-targeting peptides developed to date, emphasizing their efficacy as targeting ligands and outlining their advantages and disadvantages when compared to alternative targeting agents. Our discussion extends to their applications in both preclinical and clinical studies, whether labeled with common imaging radionuclides for positron emission tomography (PET) and single-photon emission computed tomography (SPECT) or with therapeutic radionuclides for cancer radionuclide therapy. Lastly, we assess the challenges associated with translating these radiolabeled peptides to clinical use and contemplate the prospects for future research in this field.

## 2. EGFR and Integrin α_V_β_3_ as Cancer Targets

The EGFR, also called ERBB-1 (Erythroblastic Leukemia Viral Oncogene Homologue-1) or HER-1 (Human Epidermal Growth Factor Receptor-1), is a 170 kDa single-chain transmembrane glycoprotein belonging to the ERBB family of receptor tyrosine kinases. EGFR presents three domains: (a) a hydrophobic transmembrane domain, which is associated with dimerization interaction among receptors; (b) an intracellular tyrosine kinase domain responsible for substrate phosphorylation; and (c) an extracellular EGFR-binding domain (sEGFR), which binds ligands which stimulate EGFR [5,6]. The sEGFR consists of four domains (I, II, III and IV), and exhibits two conformations: an open conformation when bound to a ligand and a closed, auto-inhibited conformation [1]. Ligand binding induces conformational changes, exposing a dimerization arm that facilitates the formation of homodimers and heterodimers with other ERBB family members. This dimerization activates the kinase domain, which consists of two lobes, with a cleft between them containing four critical structural sites: (1) the catalytic region, (2) the hinge site, (3) the activation loop, and (4) the kinase specificity pocket [7,8,9,10]. 

The aberrant activation of those EGFR signaling pathways, due to factors like cell-surface overexpression, autocrine activation, and mutations in the EGFR gene, is observed in various cancers, including lung, head and neck, cervical, colorectal, and brain cancers [11]. Among the various EGFR mutations, the most prevalent is EGFRvIII, an oncogene formed by a large extracellular deletion [12]. 

While EGFR itself is a critical target in cancer therapy due to its overexpression and mutations in various tumors, it is essential to recognize that the downstream signaling pathways activated by EGFR are the primary drivers of cancer progression. EGFR activation triggers two key signaling cascades in cancer: the PI3K/AKT/mTOR and MAPK pathways. The PI3K/AKT/mTOR pathway regulates cell growth, proliferation, and survival [13,14], while the MAPK pathway controls gene expression and cell cycle progression [11,15]. Both pathways are frequently dysregulated in cancers, contributing to uncontrolled growth and therapy resistance. NF-κβ [16] and GTPase path signaling are also activated [17]. The result of these intracellular pathways is the increased transcription of genes responsible for cell growth, survival, and/or migration [18]. Significant crosstalk between these pathways can lead to compensatory mechanisms, making single-pathway inhibition often ineffective. Understanding these interactions is crucial for developing combination therapies that target multiple pathways simultaneously to overcome resistance and improve cancer treatment outcomes.

On the other hand, integrins are a family of transmembrane cell surface receptors that mediate cell–matrix and cell–cell interactions [19]. These heterodimeric glycoproteins consist of noncovalently connected α and β subunits. In mammals, 18 α and 8 β subunits combine to form at least 24 distinct integrin heterodimers [20,21]. Each integrin subunit has a large extracellular domain (750–1000 residues), a single transmembrane domain, and a short cytoplasmatic domain [22,23]. Integrins bind to specific endogenous ligands, including cell surface counter-receptors, soluble ligands, and extracellular matrix (ECM) proteins [24]. Eight integrin heterodimers can recognize RGD-containing peptides [25]. Ligand binding triggers interactions between the cytoplasmatic domain, intracellular proteins, and cytoskeletal filaments, initiating signaling pathways through the Src kinases, which are activated by FAK phosphorylation [26]. This signaling controls key cellular functions, including adhesion, migration, proliferation, differentiation, and survival, that are critical for tissue homeostasis, development, and repair [27]. Integrin dysregulation is involved in the pathogenesis of several diseases characterized by altered angiogenesis, inflammation, or infection [28].

Integrin α_V_β_3_, also known as the vitronectin receptor, is of particular interest in cancer research. While α_V_β_3_ is expressed at low levels or is undetectable in most healthy adult epithelia, it is overexpressed in various tumor cells (breast, lung, glioblastoma, prostate, and melanoma), the tumor-associated vasculature, and invasive tumor fronts [23]. Interactions between α_V_β_3_ and its ECM ligands activate intracellular signaling pathways like PI3K/Akt and ERK/MAPK, promoting cancer progression. The engagement of α_V_β_3_ with the ECM also facilitates crosstalk with growth factor receptors and proteases to further enhance tumor cell survival and motility [29]. Notably, α_V_β_3_ expression is associated with the acquisition of a cancer stem cell phenotype, conferring tumors with enhanced initiation and therapy resistance capabilities [30]. 

Therefore, targeting EGFR and/or integrin α_V_β_3_ with specific inhibitors or antibodies/small molecules can disrupt these signaling pathways, inhibiting tumor growth and spread.

## 3. EGFR- and Integrin α_v_β_3_-Targeting Peptides

### 3.1. EGFR-Targeting Peptides

Several EGFR-targeting peptides have been identified and developed as potential diagnostic and therapeutic agents. EGFR peptides can be obtained from experimental synthesis, phage display libraries, computer-aided design, and natural sources. The most widely explored peptide is GE11 [31,32,33,34,35,36,37,38]. GE11 is a dodecapeptide that is identified through phage display screening. It exhibits high affinity and selectivity for EGFR. It binds to EGFR with a dissociation constant (Kd) of 22 nM, which is lower than that of the natural ligand EGF (Kd = 2 nM) but significantly higher than seen for non-specific binding to proteins like bovine serum albumin. This lower affinity compared to EGF is attributed to GE11’s smaller size and its binding to only one EGFR region, in contrast to EGF’s interaction with multiple domains [18]. De Paiva et al. [39] identified the binding site between GE11 and sEGFR using molecular dynamics and molecular docking simulation. According to these researchers, GE11 acts as an inhibitor. The optimal conformation of GE11 and sEGFR occurs at domains II and IV, which may block the exposure of the dimerization arm and prevent dimer formation [1,40]. Importantly, while GE11 binds to EGFR with high specificity, it does not exhibit mitogenic activity. This characteristic makes it an attractive option for targeted drug delivery without stimulating tumor growth. Recent studies have shown that GE11 can enhance nanoparticle endocytosis through an alternative EGFR-dependent, actin-driven pathway [18]. This mechanism allows for the maintenance of EGFR levels on the cell surface after GE11 binding, potentially enabling prolonged receptivity to GE11-conjugated therapeutics. Other peptides like EBP (CMYIEALDKYAC) and D4 (LARLLT) have also shown promise in EGFR targeting. EBP was experimentally synthesized and demonstrated high affinity for EGFR, while D4 was developed through computer-aided design approaches [1]. Recent research by Tripathi et al. (2024) [16] demonstrated the anticancer potential of short peptides, derived from the conserved regions of the MIEN1 protein. Their study highlighted that a six-amino-acid peptide, LA3IK, effectively inhibited EGF-mediated NF-kB nuclear translocation in breast cancer cells. This finding underscores the therapeutic promise of targeting the MIEN1 signaling pathway to impede cancer progression. 

In addition to targeting wild-type EGFR, there has been significant progress in developing peptides that are specific to EGFR mutations, such as EGFRvIII, which is commonly associated with aggressive cancers like glioblastoma. EGFRvIII, characterized by the deletion of exons 2–7, leads to a constitutively active receptor that drives tumorigenesis. Recent studies have utilized phage display technology to identify cyclic peptides that selectively bind to EGFRvIII. For instance, novel cyclic peptides P6 and P9 have shown high specificity for EGFRvIII-expressing cells, enhancing targeted drug delivery and cytotoxicity in non-small-cell lung cancer (NSCLC) and glioblastoma models [41]. These advancements underscore the potential of peptide-based therapies to effectively target specific EGFR mutations, offering new avenues for precision oncology.

These peptides, along with others listed in Table 1, represent a diverse array of EGFR-targeting strategies, each with unique binding properties and potential applications in cancer diagnostics and therapeutics.

### 3.2. Integrin α_v_β_3_-Targeting Peptides

Peptides containing the Arg-Gly-Asp (RGD) motif are the most studied α_V_β_3_ ligands [19]. Table 2 summarizes some of the integrin α_V_β_3_-targeting peptides explored in cancer research.

## 4. Radiolabeled Peptides as Valuable Tools for Imaging and the Treatment of Cancer

The process of radiolabeling peptides involves attaching radioactive isotopes to peptides, which can then be detected using imaging techniques to provide real-time, non-invasive insights into the molecular environment of tumors. This capability not only aids in the accurate diagnosis and staging of cancer but also facilitates the monitoring of treatment responses and the detection of metastases. Furthermore, when these radiolabeled peptides are designed to carry therapeutic radionuclides, they serve a dual function by also providing targeted radionuclide therapy, delivering cytotoxic radiation directly to tumor cells and thereby reducing the tumor burden while sparing normal tissues [60]. 

### 4.1. Strategies for Radiolabeling Peptides 

**Figure 1:**
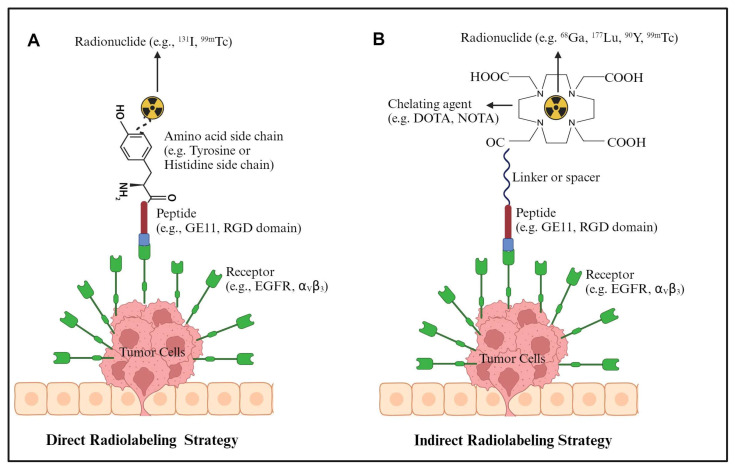
Radiolabeling strategies: (**A**) direct (**B**) indirect.

Peptide radiolabeling employs two primary methods: direct and indirect labeling (Figure 1).

#### 4.1.1. Direct Labeling

The radioisotope is covalently attached to the peptide. This is commonly performed with radioiodines like iodine-125 (^125^I, t_1/2_ = 59.4 days; Eγ = 35 keV) and iodine-131 (^131^I, t_1/2_ = 8 days; 90% β^−^ = 606 keV) via the electrophilic radioiodination of the tyrosine side chain aromatic ring. Oxidizing agents such as Chloramine T or Iodo-Gen^®^ are used to convert iodide into an electrophilic iodate that is substituted onto the tyrosine’s aromatic ring at room temperature [61]. This procedure offers the advantage of not modifying the amino acid sequence. Examples of direct iodination include ^125^I-labeling of GE11 [31] and ^131^I labeling of GRGDYV [62]. 

Direct ^99m^Tc- (t_1/2_ = 6 h; Eγ = 140 keV) labeling is also performed for peptides with disulfide bonds or via the formation of a [^99m^Tc(CO)_3_]^+^ complex that binds the histidine side chain imidazole ring. This two-step approach first makes the [^99m^Tc(H_2_O)_3_(CO)_3_]^+^ core, which then labels the histidine to form a stable complex, as demonstrated for GRGDHV [62]. The study of Baishya and coworkers evaluated two [^99m^Tc(CO)_3_]^+^-labeled tetrapeptides and one [^99m^Tc(CO)_3_]^+^-labeled hexapeptide by changing the amino acid sequence of the RGD motif for potential use as tumor-targeting radiopharmaceuticals [63]. Comparative in vivo studies of [^99m^Tc(CO)_3_]^+^-labeled PEGylated and non-PEGylated cRGDfK demonstrated that the addition of a PEG_7_ unit increased the melanoma tumor uptake and slowed the clearance from other organs, decreasing target-to-background ratios [64]. However, ^99m^Tc peptide labeling more often uses indirect methods.

#### 4.1.2. Indirect Labeling

The bifunctional chelator or prosthetic group is attached to the peptide, which then complexes with the radiometal. Typically, the chelator is coupled to the peptide first to simplify the radiosynthesis. Linkers can also be added between the chelator and peptide [65].

For integrin α_V_β_3_ imaging, ^18^F (t_1/2_ = 109.7 min; 97% β^+^; Eβ^+^_max_ = 635 keV) has been used most, followed by ^68^Ga and ^64^Cu. ^18^F-Galacto-RGD was the first RGD PET tracer in humans [66,67], and it was made by conjugating a sugar amino acid to c(RGDfK) and labeling with ^18^F-fluoropropionate. This radiolabeling process is time-consuming, which presents a hurdle for large-scale clinical studies. Similar challenges are observed when labeling other tracers like ^18^F-FPPRGD and ^18^F-RGD [25,66,67,68,69,70]. Other ^18^F-RGD tracers, such as ^18^F-FPPRGD and ^18^F-Alfatide I/II, provide a more efficient production process with easier and faster radiosynthesis (40 min and 20 min for kit radiolabeling) and higher yields (42%, radiochemical purity > 95%) [69]. 

As an alternative to ^18^F in peptide labeling, ^68^Ga presents advantageous physical characteristics. ^68^Ga (t_1/2_ = 68 min; 89% β^+^; Eβ^+^_max_ = 1920 keV) can be eluted from an in-house ^68^Ge/^68^Ga generator and its half-life aligns well with the pharmacokinetics of several peptides. Many chelators, such as DOTA, NODAGA, NOTA, 1,4,7-triazacyclononane-1,4,7-tris[(2-carboxyethyl)methylenephosphinic acid (TRAP), and tris-hydroxypyridinone (THP), form stable complexes with ^68^Ga, allowing for labeling in short reaction times, often at room temperature [25,69,71,72,73]. A study conducted by Lang et al. (2011) demonstrated that, for the same chelator–peptide complex (NOTA-PRGD2), the labeling yield required to form a ^68^Ga complex was higher than that needed to form a ^18^FAl complex [67,74]. 

^64^Cu (t_1/2_ = 12.7 h; 19% β^+^, Eβ^+^_max_ = 656 keV; 38% β^−^, Eβ^−^_max_ = 573 keV; 43% EC) also stands out as an attractive radionuclide for PET imaging because of its favorable decay characteristics, coupled with the ability to produce it at high specific activity levels on small biomedical cyclotrons [75]. 

Concerning EGFR-targeting peptides, most radiolabeling approaches were performed using ^99m^Tc [33,34,76,77,78]. 

Over 80% of the radiopharmaceuticals utilized for SPECT imaging rely on ^99m^Tc-labeled compounds due to their favorable nuclear properties and widespread availability through ^99^Mo/^99m^Tc generators at a low cost. With a half-life of 6 h, ^99m^Tc allows radiopharmacists ample time for radiosynthesis, while still permitting physicians to obtain clinically pertinent images. Among the chelating agents employed for the labeling of ^99m^Tc compounds, HYNIC (6-hydrazinonicotinic acid) is the most extensively utilized [33,76,78].

Table 3, Table 4, Table 5 and Table 6 offer an overview of the different radiolabeling strategies used for EGFR- and integrin α_V_β_3_-targeting peptides.

## 5. Preclinical Studies of Radiolabeled EGFR and Integrin α_v_β_3_-Targeting Peptides 

### 5.1. EGFR-Targeting Peptides

**Figure 2:**
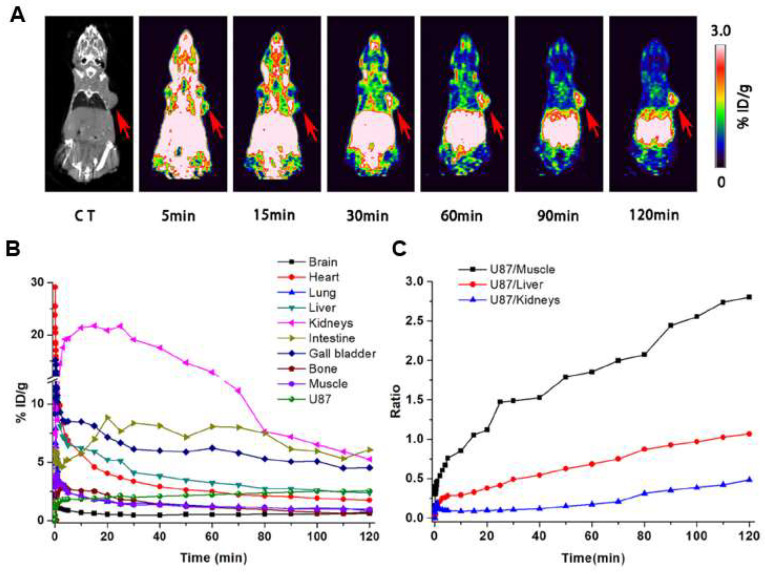
Preclinical study using radiolabeled GE11 for imaging of EGFR-positive tumors (**A**) Dynamic PET/CT images of [^18^F]F-FP-Lys-GE11 from U-87 MG tumor-bearing mice at 5, 15, 30, 60, 90, and 120 min p.i. The red arrow indicates tumor. (**B**) Time–activity curves. (**C**) Tumor/organ ratios (tumor/muscle, tumor/liver, tumor/kidney). Reproduced with permission from reference [32].

The GE11 peptide has been labeled with various radionuclides, including ^18^F [32,79], ^111^In [79], ^64^Cu [36,81,132], ^99m^Tc [33,34,37], and ^68^Ga [82,133,134,135]. However, its EGFR-targeting efficacy is controversial. Some studies reported good binding affinity and tumor uptake in cell lines and murine models [31,32,33,34,37]. Figure 2 illustrates dynamic PET/CT images of [^18^F]F-FP-Lys-GE11 from U-87 MG tumor-bearing mice, time–activity curves, and tumor/organ rates.

Other studies cast doubt on GE11’s EGFR affinity [36,82]. Striese et al. [36,82] and Judmann et al. [36,82] attributed this lack of targeting efficacy to GE11’s high hydrophobicity, which may cause peptide aggregation and limit its interactions with EGFR. They also proposed that the cell uptake reported in other GE11 studies may be facilitated by highly hydrophilic linkers or constructs with multiple peptide copies, suggesting that developing small-molecule GE11-based radioligands may not be a promising approach to obtaining alternatives to GE11. Few attempts have been made in terms of other peptide scaffolds [50,76,77,78,83]. Table 7 summarizes all the collected preclinical data.

#### Use of EGFR-Targeting Peptides for Therapeutic Purposes

No studies have yet been conducted that utilize radiotherapeutic isotopes for the treatment of conditions via EGFR-targeting peptides. This gap in the research might be attributed to the controversial efficacy of EGFR targeting itself. This inconsistency in the efficacy of EGFR targeting could potentially prevent the development of therapeutic applications involving radiotherapeutic isotopes, as the foundational premise of specific and effective EGFR targeting remains under debate [36,82].

### 5.2. Integrin α_V_β_3_-Targeting Peptides

The development of radiotracers for imaging purposes employs various strategies [136]. The multimerization of the cRGD scaffold is an approach that leverages the polyvalence effect to increase the binding affinity to integrin α_V_β_3_. This method suggests that radiotracers derived from multimeric peptides exhibit higher tumor uptake and better tumor/background ratios than their monomeric counterparts [104,137,138]. However, this can also lead to increased non-specific uptake in non-target tissues [52]. Guo et al. [139] showed that the ^18^F-labeled RGD dimer ([^18^F]F-FP-PRGD2) had a greater binding affinity than the monomer ([^18^F]F-FP-RGD) in mice bearing MDA-MB-435 tumor xenografts (difference in %ID/g uptake: RGD2/RGD~1.5, *p* = 0.0045). Chen et al. [68] also demonstrated the higher tumor uptake of [^18^F]F-FPRGD2 than [^18^F]F-FPRGD at all time points in a glioblastoma xenograft mouse model. The dimeric tracer showed predominantly renal excretion, while the monomeric tracer was excreted primarily through the biliary route, resulting in higher tumor/background ratios [69].

Pegylation has been shown to extend the radiotracer’s circulation time and modulate its clearance, without affecting rapid elimination from the liver and kidneys [25,67]. Glycosylation, involving the conjugation of a sugar amino acid to the peptide structure, also enhances hydrophilicity and blood circulation time. One notable example of a glycosylated radiotracer is [^18^F]F-Galacto-RGD, which demonstrates significant tumor uptake, fast blood clearance, and predominantly renal elimination [25,66,140]. 

**Figure 3:**
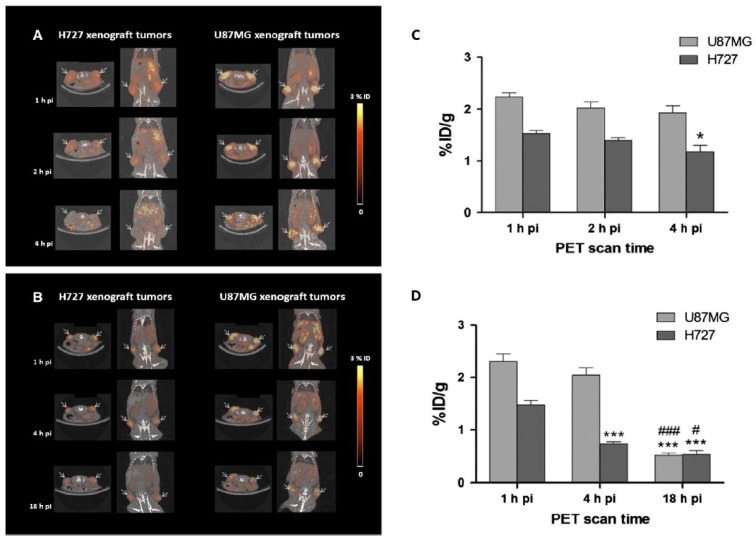
Preclinical studies using radiolabeled RGD dimer to image integrin α_V_β_3_-positive tumors. (**A**) Mice bearing U-87 MG or H727 tumors scanned with [^68^Ga]Ga-NODAGA-RGD2 at 1, 2, and 4 h p.i.; (**B**) Mice bearing U-87 MG or H727 tumors scanned with [^64^Cu]Cu-NODAGA-RGD2 at 1, 4, and 18 h p.i. Tumors are indicated by arrows; (**C**) [^68^Ga]Ga-NODAGA-RGD2 uptake in U-87 MG or H727 tumors; (**D**) [^64^Cu]Cu-NODAGA-RGD2 in U-87 MG or H727 tumors uptake. Reproduced with permission from reference [87]. * *p* < 0.05 for 1 versus 4 h p.i.; *** *p* < 0.001 for 1 versus 4 or 18 h p.i.; # *p* < 0.05 for 4 versus 18 h p.i.; ### *p* < 0.001 for 4 versus 18 h p.i.

In preclinical studies, RGD radiotracers have been evaluated in cancer models of breast cancer, lung cancer, melanoma, and especially glioblastoma. The U-87 MG cell line is the most reported among xenograft models [141]. Figure 3 illustrates the preclinical study conducted by Oxboel et al. [87], who used radiolabeled E{c(RGDFk)_2_ to image U-87 MG and H727 tumors.

Relevant preclinical studies performed with radiotracers targeting integrin α_V_β_3_ are shown in Table 8 and Table 9.

#### Use of Integrin α_V_β_3_-Targeting Peptides for Therapeutic Purposes

The radioisotopes that are mainly in use are the β^−^ emitters, like Lutetium-177 (^177^Lu) and Yttrium-90 (^90^Y) (Table 10) [125,126,127,128,129,130,131]. ^177^Lu emits both β^−^ particles and γ rays, which allows, respectively, radionuclide therapy and SPECT imaging to confirm the distribution and localization of the therapeutic agent. ^177^Lu has a half-life of 6.7 d and a tissue penetration range of about 2 mm, making it suitable for treating small- to medium-sized tumors. Conversely, ^90^Y is a pure β^−^ emitter with a higher energy and longer range (approximately 11 mm) than ^177^Lu. These characteristics make it effective in treating larger tumors. However, its shorter half-life of 2.7 d requires more precise timing in clinical applications [129,144]. The efficacy of ^177^Lu- and ^90^Y-labeled integrin α_V_β_3_-targeting peptides in animal models has shown promising results. However, some challenges need to be addressed to enhance the efficacy and safety of these therapies: the heterogeneity of tumor expression, the development of resistance, and radiation toxicity (this can affect surrounding healthy tissues) [145]. Further research is needed to explore the use of other therapeutic radioisotopes or hybrid peptides that might offer better therapeutic profiles.

## 6. Clinical Studies

Table 11 summarizes some clinical studies performed using radiolabeled RGD-based peptides for cancer imaging and therapy. These trials show promising efficacy in terms of tumor detection, staging, and monitoring treatment responses, highlighting their potential in enhancing the precision of cancer diagnostics and therapy.

## 7. Dual-Targeting Peptides

Achieving optimal single-target tumor imaging and therapy is challenging due to receptor heterogeneity, low binding affinities, and suboptimal in vivo pharmacokinetics. These limitations hinder the generation of high-quality diagnostic images and the effective application of monomeric radiopeptides [82]. The solution is the development of heterodimeric peptides (HPs) that link two distinct specific peptide ligands [160]. To date, only a few HPs for bispecific EGFR and integrin α_V_β_3_ targeting have been described [132,133,135,161], highlighting the novelty and limited availability of such agents. 

**Figure 4:**
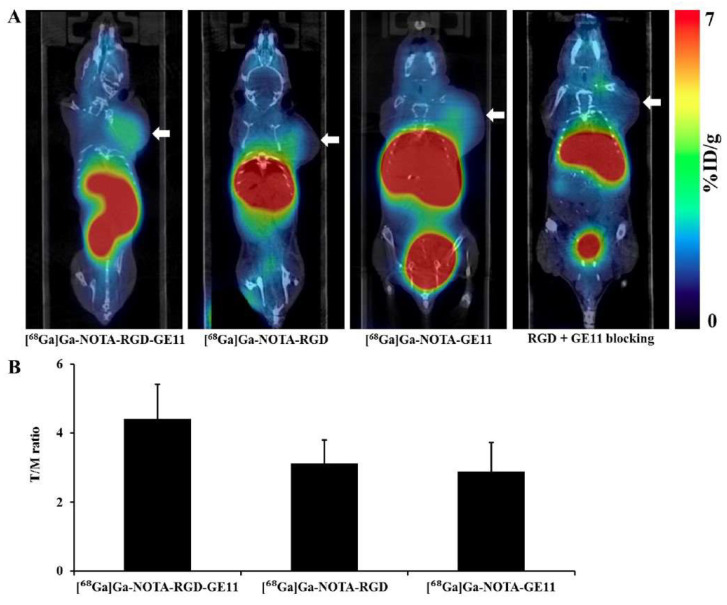
(**A**) PET/CT imaging of lung tumor-bearing mice (NCI-H292 cells) 2 h p.i. of [^68^Ga]Ga-NOTA-RGD-Cys-6-Ahx-GE11, [^68^Ga]Ga-NOTA-RGD, [^68^Ga]Ga-NOTA-GE11, [^68^Ga]Ga-NOTA-RGD-Cys-6-Ahx-GE11 + blocking. Arrows indicate tumors. (**B**) Tumor/muscle (T/M) ratio of [^68^Ga]Ga-NOTA-RGD-Cys-6-Ahx-GE11, [^68^Ga]Ga-NOTA-RGD, [^68^Ga]Ga-NOTA-GE11. Reproduced with permission from reference [161].

Yu et al. [133] designed [^68^Ga]Ga-NOTA-RGD-Cys-6-Ahx-GE11. Chen et al. [161] subsequently investigated the in vitro and in vivo properties of this radiotracer, comparing it with those of monomeric radiopeptides. The superiority of HPs was noticed in terms of binding affinities and tumor uptake in biodistribution and PET/CT imaging studies, as shown in Figure 4. Specifically, in the PET/CT imaging study at 2 h p.i., the tumor uptake values and tumor/muscle ratios were 3.5 ± 0.6%ID/g and 4.4 ± 1.0 for HPs, 2.4 ± 0.3%ID/g and 2.9 ± 0.7 for [^68^Ga]Ga-NOTA-GE11, and 2.8 ± 0.5%ID/g and 3.1 ± 0.7 for [^68^Ga]Ga-NOTA-RGD, respectively. However, in all cases, the liver and kidneys presented high activity levels, highlighting the need for further ligand structure modifications to achieve better pharmacokinetics [161,162]. 

Braun et al. [135] refined the radiotracer developed by Chen et al. [161]. They replaced the cysteine building block with (NH_2_-propyl)_2_Gly-OH to achieve a more uniform structure and used NODA as the chelator. PEG spacers were also incorporated. [^68^Ga]Ga-NODA-(PEG_3_-GE11-PEG_3_-c(RGDyK)) and [^68^Ga]Ga-NODA-(PEG_5_-GE11-PEG_5_-c(RGDfK)) were synthesized. In vitro cell (A431) uptake studies demonstrated favorable integrin α_V_β_3_-specific receptor affinities for these two bispecific agents. However, they did not exhibit receptor-specific interactions with the EGFR in the in vitro studies. These in vitro findings were corroborated by PET/CT imaging in tumor-bearing mice, which showed that the observed tumor uptake was only mediated by integrin α_V_β_3_ and not by EGFR binding [135].

**Figure 5:**
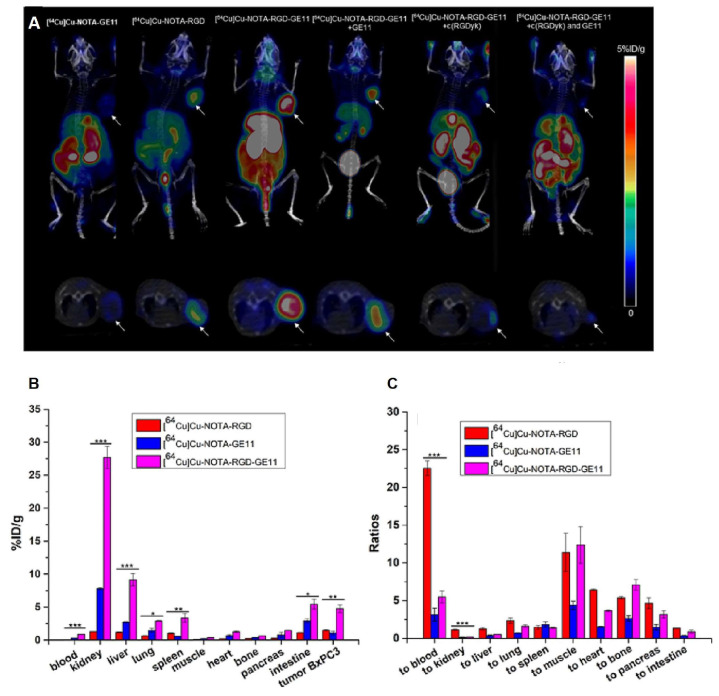
(**A**) PET/CT imaging and (**B**,**C**) biodistribution of pancreas adenocarcinoma-bearing mice (BxPC3 cells) 2 h p.i. of [^64^Cu]Cu-NOTA-GE11, [^64^Cu]Cu-NOTA-RGD, [^64^Cu]Cu-NOTA-RGD-GE11 and in the presence of blocking dose of GE11, c(RGDyk), or both unlabeled peptides. The arrows indicate the tumors. Significant differences are indicated by: * *p* < 0.05, ** *p* < 0.01 and *** *p* < 0.001. Reproduced with permission from reference [132].

In the same year, Li et al. [132] synthesized [^64^Cu]Cu-NOTA-RGD-GE11. The bispecific agent was compared to its monomeric units. [^64^Cu]Cu-NOTA-RGD-GE11 demonstrated significantly enhanced tumor uptake (4.6 ± 0.2%ID/g) compared to monomeric agents (1.2 ± 0.2%ID/g for [^64^Cu]Cu-NOTA-RGD and 0.8 ± 0.1%ID/g for [^64^Cu]Cu-NOTA-GE11) at 2 h p.i. in mice bearing BxPC3 xenografts, as shown in Figure 5. The tumor uptake of the HPs was effectively inhibited in the presence of both non-radioactive c(RGDyK) and GE11, suggesting that both peptides exhibited receptor-specific interactions with their respective targets. These findings underscore the potential of dual-targeting peptides in improving the specificity and effectiveness of cancer therapeutics and diagnostics, paving the way for future clinical applications.

## 8. Conclusions and Future Directions

Herein, we undertook a comprehensive review of the development and application of EGFR- and integrin α_V_β_3_-targeting peptides as potential radiometal-labeled radiopharmaceuticals for cancer theranostics. The use of these peptides in both diagnostic and therapeutic contexts offers a dual benefit by enabling precise tumor imaging and targeted therapy, potentially leading to better patient outcomes. 

Despite the promising aspects of peptide-based radiopharmaceuticals, several challenges and limitations persist. One major challenge is the inherent lower binding affinity and the rapid clearance from the bloodstream of peptides, which can limit their therapeutic efficacy and diagnostic accuracy. Also, the renal toxicity associated with radiometal-labeled peptides accumulation poses a significant concern for patient safety. The small size of peptides, while beneficial for tumor penetration, also contributes to their rapid degradation and clearance, necessitating frequent or higher dosages.

To overcome these challenges, several emerging strategies and technologies are being explored. One approach is the modification of peptides to enhance their stability and binding affinity. This includes the use of cyclization, PEGylation, and the incorporation of non-natural amino acids, which can improve metabolic stability and reduce renal clearance. Another strategy is the development of multivalent and multimeric peptide systems that can increase functional affinity and selectivity towards target receptors. Additionally, the exploration of alternative targeting moieties, such as small-molecule ligands or scaffold proteins, offers a potential avenue for increasing the therapeutic index of these potential radiopharmaceuticals.

The clinical translation of EGFR- and integrin α_V_β_3_-targeting peptides faces several hurdles that must be addressed in order to realize their full potential. The optimization of peptide structures to enhance receptor binding and stability, coupled with advanced radiolabeling techniques, is crucial for improving the efficacy and safety profiles of these agents. Clinical trials are essential for evaluating the therapeutic benefits, potential side effects, and overall patient outcomes associated with these novel potential radiopharmaceuticals. Furthermore, regulatory approval will be pivotal in determining the feasibility of incorporating these targeted therapies into standard clinical practice.

While EGFR- and integrin α_V_β_3_-targeting peptides hold significant promise for enhancing cancer diagnosis and treatment, extensive research and development are still required to address the existing challenges. With continued advancements in peptide engineering and radiolabeling technologies, these agents can become integral components of precision oncology, offering more effective and personalized treatment options for cancer patients. In addition, while the targeting of EGFR and integrin is a valid therapeutic strategy, it is the downstream signaling pathways, particularly PI3K/AKT/mTOR and MAPK, that drive cancer progression and therapeutic resistance. Future research and therapeutic development should focus on these pathways to achieve more effective cancer treatments. By integrating inhibitors of these pathways with EGFR-targeted therapies, it may be possible to enhance treatment efficacy and overcome resistance mechanisms, leading to better patient outcomes. 

## Figures and Tables

**Table 1:** Examples of EGFR-targeting peptides.

Name	Sequence	Source	Ref.
EBP	CMYIEALDKYAC	Experimentally synthesized.	[42]
S3	RCSHGYTGIRCQAVVL	From the third loop structure and linear C-terminal region of vaccinia virus growth factor (VGF).	[43,44]
GE11	YHWYGYTPQNVI	Screened out from a phage display peptide library.	[18,31]
P1	SYPIPDT		[45]
P2	HTSDQTN		[45]
QRH	QRHKPRE		[46]
D4	LARLLT	Computer-aided design approach.	[47]
P75	KYFPPLALYNPTEYFY	From one-bead-one-compound (OBOC) library.	[48]
Pep 11	WSGENGPGFYDYEA	Designed using knob-socket model.	[49]
---	EEEEYFELV DEDEYFELV	Experimentally synthesized.	[50]

**Table 2:** Examples of integrin α_V_β_3_-targeting peptides.

Examples	Comments
**RGD peptides**	
**RGD, c(RGDfK), c(RGDyK)**	RGD peptides can present linear or cyclic formats (cRGD) with different levels of selectivity and specificity. Linear RGD peptides are more sensitive to chemical degradation [51]. Cyclization aims to decrease susceptibility to enzymatic degradation, enhancing tracer stability [52]. c(RGDyK) and c(RGDfK) have been extensively studied, with c(RGDyK) showing superior affinity for integrin α_V_β_3_ [25].
**iRGD**	iRGD is a 9-amino-acid cRGD derived from phage display screening [53].
**LXW7 and LXW64**	LXW7 (cGRGDdvc-NH_2_) was derived from the OBOC library and LXW64 (cGRGDd-DNaI1-c-NH_2_) is its optimized version [54].
**RGD-4C and NC-100717**	RGD-4 C is a double-cysteine-bridged peptide and the development of NC-100717 was based on the PEGylation of RGD-4C [51,55,56].
**c(RGDf[NMe]V (cilenglitide) and RGDechi**	Cilenglitide acts as a selective α_V_β_3_ antagonist. Preclinical studies have demonstrated its antiangiogenic and antitumor effects in various cancer models [45]. While cilengitide was well tolerated in phase I/II clinical trials [46,47], it failed to show efficacy in phase III studies [48], likely due to the complexity and plasticity of integrin signaling networks. RGDechi is a designed α_V_β_3_ antagonist based on cilenglitide structures combined with echistatin C-terminal tails [57].
**Non-RGD peptides**	
ATN-161	ATN-161 (Ac-PHSCN-NH_2_) is a capped pentapeptide synthesized from the fibronectin—PHSRN sequence and it can be used alone or along with radiotherapy and chemotherapy to prevent metastasis and tumor development [49,50,51]. In phase I of a clinical study, ATN-161 was used for aggressive solid tumors and demonstrated good toleration and safety [53].
**RWrNK and RWrNM**	RWrNK and RWrNM are linear peptides that contains an unnatural d-arginine (r). They present great water solubility and the ability to pass through the blood–brain tumor barrier. They have been investigated for glioblastoma diagnosis [58,59].

**Table 3:** Direct labeling strategies for EGFR- and integrin α_V_β_3_-targeting peptides.

EGFR-Targeting					
Radionuclide	Peptide/Sequence	Formulation	RCY * (%)	RCP ** (%)	Ref.
^124^I	GE11	[^124^I]I-GE11			[31,35]
		[^124^I]I-GE11	≥47	≥98	[79]
^131^I	EEEEYFELV	[^131^I]I-EEEEYFELV	>84	>90	[50]
	DEDEYFELV	[^131^I]I-DEDEYFELV	>91	>90	
**Integrin α_V_β_3_-Targeting**					
^125^I	c(RGDyK)	[^125^I]I-c(RGDyK)	≥89	>95	[80]
^77^Br		[^77^Br]Br-c(RGDyK)	≥73	>95	
^131^I	GRGDYV	[^131^I]I-GRGDYV	>95	>94	[62]
^99m^Tc	GRGDHV	[^99m^Tc]Tc(CO)_3_-GRGDHV	>95	>94	

* RCY: radiochemical yield; ** CP: radiochemical purity.

**Table 4:** Indirect labeling strategies for EGFR-targeting peptides.

EGFR-Targeting							
Radionuclide	Chelator/ Prosthetic Group	Linker/Spacer	Peptide	Formulation	RCY * (%)	RCP ** (%)	Ref.
^64^Cu	p-SCN-Bn-NOTA		GE11	[^64^Cu]Cu-NOTA-GE11	46	90	[81]
		β-alanina + ethylene glycol-based linker		[^64^Cu]Cu-NOTA-linker-β-Ala-GE11		>99	[36]
				[^64^Cu]Cu-NOTA-linker-β-Ala-GE11-NH_2_		>99	
^18^F	N-succinimidyl 4-fluorobenzoate (SFB)	-Gly-Gly-Gly-Lys-(GGGK)		[^18^F]F-SFB-GGGK-GE11		95	[79]
	F-PEG_4_-propyne			[^18^F]F-F-PEG_4_-propyne-GGGK-GE11		98	
	4-nitrophenyl-2-fluoropropionate (NFP)		GE11/GE11-Lys	[^18^F]F-NFP-Lys-GE11	7	>99	[32]
^68^Ga	NODAGA	PEG (different lengths)	GE11	[^68^Ga]Ga-NODAGA-PEG_n_-GE11		≥97	[82]
		PEG_5_		[^68^Ga]Ga-NODAGA-PEG_5_-GE11	5–74	≥98	[38]
			D4	[^68^Ga]Ga-NODAGA-PEG_5_-D4			
			P1	[^68^Ga]-NODAGA-PEG_5_-P1			
			P2	[^68^Ga]Ga-NODAGA-PEG_5_-P2			
			CPP	[^68^Ga]Ga-NODAGA-PEG_5_-CPP			
			EGBP	[^68^Ga]Ga-NODAGA-PEG_5_-EGPB			
			QRH	[^68^Ga]Ga-NODAGA-PEG_5_-QRH			
			Pep11	[^68^Ga]Ga-NODAGA-PEG_5_-Pep11			
^124^I	N-succinimidyl 4-iodobenzoate (SIB)	GGGK	GE11	[^124^I]I-SIB-GGGK-GE11	30	98	[79]
^111^In	p-SCN-Bn-NOTA			[^111^In]In-NOTA-GGGK-GE11		100	
^99m^Tc	HYNIC + tricine	Seryl-seryl-serine residue (SSS)		[^99m^Tc]Tc-tricine-HYNIC-SSS-GE11	>98	95	[34]
			D4	[^99m^Tc]Tc-tricine-HYNIC-SSS-D4	98	98	[78]
				[^99m^Tc]Tc-tricine-HYNIC-D4	98	98	[77]
	HYNIC + tricine + EDDA	SSS		[^99m^Tc]Tc-tricine/EDDA-HYNIC-SSS-D4		98	[76]
			GE11	[^99m^Tc]Tc-tricine/EDDA-HYNIC-SSS-GE11	>99	99	[33]
	-Gly-Gly-Gly-Cys-(GGGC)			[^99m^Tc]Tc-GGGC-GE11	>98	>90	[37]
	ECG (tripeptide–several nitrogen atoms and one sulfur atom)	Histidine-containing spacer peptide (GHEG)	P1	[^99m^Tc]Tc-ECG-SYPIPDT-ECG-TAMRA	>95		[83]

* RCY: radiochemical yield; ** RCP: radiochemical purity.

**Table 5:** Indirect labeling strategies for integrin α_V_β_3_-targeting peptides.

Radionuclide	Chelator/Prosthetic Group	Linker/Spacer	Peptide	Formulation	RCY * (%)	RCP ** (%)	Ref.
^64^Cu	DOTA		E{E[c(RGDfK)]_2_)_2_	[^64^Cu]Cu-DOTA-E{E[c(RGDfK)]_2_)_2_/[^64^Cu]Cu-DOTA-RGD2	75	95	[84]
	CB-TE_2_A (4,11-bis(carboxymethyl)-1,4,8,11-tetraazabicyclo[6.6.2] hexadecane))		c(RGDyK)	[^64^Cu]Cu-CB-TE_2_A-c(RGDyK)		95	[75]
	Hexaazamacrobicyclic sarcophagine (Sar) based chelator (diamSar)		c(RGDfD)	[^64^Cu]Cu-diamSar-c(RGDfD)		95	
	DOTA	PEG_4_	E[c(RGDfK)]_2_	[^64^Cu]Cu-DOTA-PEG_4_-E[c(RGDfK)]_2_	95	95	[85]
	DOTA	Triglycine (3G)	E[c(RGDfK)]_2_	[^64^Cu]Cu-DOTA-G_3_-E[c(RGDfK)]_2_	95	95	
	Sar-based chelator (BaBaSar)		E[c(RGDfK)]_2_	[^64^Cu]Cu-BaBaSar-E[c(RGDfK)]_2_/[^64^Cu]Cu-BaBaSar-RGD2	95	99	[86]
	NODAGA		E[c(RGDfK)]_2_	[^64^Cu]Cu-NODAGA-E[c(RGDyK)]_2_/[^64^Cu]Cu-RGD2	50	>89	[69,87]
	Sar-based chelator (AmBaSar)		E[c(RGDfK)]_2_	[^64^Cu]Cu-AmBaSar-RGD2/[^64^Cu]Cu-AmBaSar-E[c(RGDfK)]_2_	>90	98	[88]
	NOTA	PEG_4_-SAA_4_	c(RGDfK)	[^64^Cu]Cu-NOTA-PEG_4_-SAA_4_-c(RGDfK)	>90		[52]
	NOTA	PEG_2_	c(RGDfK)	[^64^Cu]Cu-NOTA-PEG_2_-c(RGDfK)	>90		
^18^F	NFP	PEG_3_	E{c(RGDyk)_2_	[^18^F]F-FPPRGD2	>73	99	[70,89,90,91]
	NFP	PEG_2_, SAA and 1,2,3-triazole	c(RGDfK)	[^18^F]F-Galacto-RGD/[^18^F]F P-SAA-RGD	>24	98	[66]
	p-fluorobenzaldeyde		aminooxy-bearing RGD peptide (AH111585)	[^18^F]F-fluciclatide/[^18^F]F-AH111585	>17	95	[92,93]
	pentyne tosylate		c(RGDyK)	[^18^F]F-RGD-K5	35	95	[94,95,96]
	SFB		E[*c*(RGDyK)]_2_	[^18^F]F-FB-RGD2	>20	99	[68]
	SFB		c(RGDyK)	[^18^F]F-FB-RGD	>35	99	[68]
	Al(NOTA)	PEG_3_	E[*c*(RGDyK)]_2_	[^18^F]F-AlF-NOTA-PRGD_2_ [^18^F]F-AlF-NOTA-PEG_3_-E[c(RGDyK)]_2_/[^18^F]F-Altatide I	>40	95	[97]
	Al(NOTA)	PEG_4_	E[c(RGDfk)]_2_	[^18^F]F-NOTA-E[PEG_4_-c(RGDfk)]_2_/[^18^F]F-AlF-NOTA-2P-RGD2/[^18^F]F-Alfatide II	>40	95	[98]
^68^Ga	NODAGA		c(RGDfK)	[^68^Ga]Ga-RGD/[^68^Ga]Ga-NODAGA-c(RGDfK)	96	98	[71,99]
	NODAGA		E[c(RGDfK)]_2_	[^68^Ga]Ga-NODAGA-E[c(RGDyK)]_2_/[^68^Ga]Ga-RGD2	50	>89	[87,100,101]
	DOTA	PEG_4_	E[c(RGDfK)]_2_	[^68^Ga]Ga-3PRGD2/[^68^Ga]Ga (PEG_4_-E[PEG_4_-c(RGDfK)]_2_).	90	98	[71,102]
	TRAP	PEG_4_	c(RGDfK)_3_	[^68^Ga]Ga-TRAP-(RGD)_3_	≥95	99	[71,103]
	p-SCN-Bn-NOTA	3 PEG_4_	E[c(RGDfK)]_2_	[^68^Ga]Ga-NOTA-PRGD_2_/[^68^Ga]Ga NOTA-PEG_4_-E[c(RGDfK)]_2_	42	95	[69,104]
	p-SCN-Bn-NOTA	3G	E[c(RGDfK)]_2_	[^68^Ga]Ga-NOTA-E[G_3_c(RGDfK)]_2_	43	95	[69,104]
	p-SCN-Bn-NOTA		c(RGDyK)	[^68^Ga]Ga-NOTA-RGD	98	99.5	[67,73]
	Fusarinine-C (FSC)		c(RGDfK)_3_	[^68^Ga]GaFSC-(RGD)_3_	94	95	[105]
	THP		c(RGDfK)	[^68^Ga]Ga-HP3-(RGD)_3_	>86	95	[72]
^125^I	SIB		c(RGDfK)	[^124^I]I-bcRGD	36	99	[106]
^99m^Tc	HYNIC + tricine +TPPTS	3G	E{c(RGDFk)_2_	[^99m^Tc]Tc-HYNIC-3G-(RGD)_2_	>60	>95	[107,108]
	HYNIC + tricine +TPPTS	3 PEG_4_	E{c(RGDFk)_2_	[^99m^Tc]Tc-3PRGD2/[^99m^Tc]Tc-HYNIC-PEG_4_-E[PEG_4_-c(RGDfK)]_2_	>95	>95	[109,110,111]
	Cysteine (Cys)	PNP = [(CH_3_)_2_P(CH_2_)_2_N(C_2_H_4_OCH_3_)(CH_2_)_2_P(CH_3_)_2_]	RGDechi	[^99m^Tc]Tc(RGDechi-Cys)PNP_43_	>82	98	[112]
	HYNIC + tricine +EDDA		c(RGDyK)	[^99m^Tc]Tc-HYNIC-RGD	>94	>95	[113]
	DOTA		RAFT-E[c(RGDfK)]_2_	[^99m^Tc]Tc-RAFT-RGD		>90	[114,115]
	HYNIC + tricine +TPPTS		E[c(RGDfK)]_2_	[^99m^Tc]Tc(HYNIC-E E[c(RGDfK) _2_]_2_)(tricine)(TPPTS)]	65	95	[116]
	MAG_2_	3G	E{c(RGDFk)_2_	[^99m^Tc]TcO(MAG_2_-3G_3_-E{c(RGDFk)_2_		95	[117]
	HYNIC + tricine +TPPTS	PEG_2_, SAA and 1,2,3-triazole	E{c(RGDFk)_2_	[^99m^Tc]Tc-Galacto-RGD2		>95	[118,119]
^111^In	DOTA		E-[c(RGDfK)]_2_	[^111^In]In-RGD2/[^111^In]InDOTA-E-[c(RGDfK)]_2_		>95	[120]
	DOTA		c(RGDfK)	[^111^In]In-RGD		90	[121]
	DOTA	PEG_2_, SAA and 1,2,3-triazole	E-[c(RGDfK)]_2_	[^111^In]In-DOTA-Galacto-RGD2		95	[122]
	DOTA	PEG_4_	E-[c(RGDfK)]_2_	[^111^In]In-3P-RGD2	33	>95	[122]
	DTPA	PEG_4_	E-[c(RGDfK)]_2_	[^111^In]In-DTPA-3PRGD2	37	>95	[123]
	DOTA		E-[c(RGDfK)]_2_	[^111^In]In-DOTA-RGD2	>95		[124]

* RCY: radiochemical yield; ** RCP: radiochemical purity.

**Table 6:** Radiolabeling strategies for α_V_β_3_-targeting peptides aiming therapy.

Radionuclide	Chelator	Linker	Peptide	Formulation	Radiolabeling Efficiency (%)	Ref.
^177^Lu	DOTA		E-c(RGDfK)	[^177^Lu]Lu-DOTA-E-c(RGDfK)	>93	[125]
	NOTA-SCN		c(RGDyK)	[^177^Lu]Lu-NOTA-SCN-c(RGDyK)	99	[126]
	DOTA	3 PEG_4_	E[c(RGDfK)]_2_	[^177^Lu]Lu-DOTA-(PEG_4_)_3_-E[c(RGDfK)]_2_/ [^177^Lu]Lu-3PRGD2	>99	[127]
^90^Y			RAFT(c[-RGDfK-])_4_	[^90^Y]Y-DOTA-RAFT-(c[-RGDfK-])_4_/[^90^Y]Y-RAFT-RGD	>90	[128]
^177^Lu				[^177^Lu]Lu-DOTA-RAFT-(c[-RGDfK-])_4_/ [^177^Lu]Lu -RAFT-RGD	>97	
	5p-C-NETA		c(RGDyK)	[^177^Lu]Lu-5p-C-NETA-c(RGDyK)	99	[129]
^90^Y				[^90^Y]Y-5p-C-NETA-c(RGDyK)	99	
	NOTA	Evans blue (EB)	c(RGDfK)	[^90^Y]Y-NOTA-EB- c(RGDfK)/[^90^Y]Y-NMEB-RGD	>90	[130]
^177^Lu				[^177^Lu]Lu-NOTA-c(RGDfK)/[^177^Lu]Lu -RGD	>95	[131]
		EB		[^177^Lu]Lu-NOTA-EB-c(RGDfK)/[^177^Lu]Lu-EB-RGD	>95	
^225^Ac	DOTA		E[c(RGDfK)]_2_	[^225^Ac]Ac-DOTA- E[c(RGDfK)]_2_/[^225^Ac]Ac-DOTA-RGD2	>95	[124]

**Table 7:** Preclinical studies performed with radiotracers targeting EGFR.

Formulation	Cell Line	In Vitro Investigation	Animal Model	In Vivo Investigation	Biodistribution Tumor Uptake (%ID/g)	Imaging Tumor Uptake	Ref.
[^124^I]I-GE11	SMMC-7721	Binding affinity, internalization, MTT	Hepatocellular carcinoma	Biodistribution (at 0.5 and 4 h p.i.), gene delivery and transfection	3.2 (4 h p.i.)	N/A	[31]
[^124^I]I -GE11	A431	Binding affinity (no inhibition)	Epidermal and glioblastoma	Efficiency of treatment	N/A	N/A	[35]
[^64^Cu]Cu-NOTA-linker-β-Ala-GE11-NH_2_ and [^64^Cu]Cu-NOTA-linker-β-Ala-GE11	A431, MDA-MB-435 and FaDu	Human serum stability, cell medium and buffer viability, binding saturation, displacement assays on intact cells and homogenates, immunoblotting	Head and neck squamous cell carcinoma (HNSC)	Biodistribution (at 5 and 60 min p.i.), PET (at 1 h and 36 h p.i.)	<1 (5 min p.i.)	Some [^64^Cu]Cu-NOTA-linker-β-Ala-GE11 accumulation was detected 5 min p.i.	[36]
[^18^F]FP-Lys-GE11	A431, U87MGand PC3	Receptor binding, partition coefficient, stability, cell uptake and blocking assays	Glioblastoma and prostate cancer	metabolism, immunohistochemistry, biodistribution at 2 h p.i., and 2 h dynamic PET/CT imaging	N/A	3.5 ± 0.4 (U87) and 3.7 ± 0.8 (PC-3)	[32]
[^99m^Tc]Tc-tricine-HYNIC-SSS-GE11	SKOV3	Plasma stability, receptor binding, internalization and, blocking assay	Ovarian cancer	Biodistribution at 1 and, 4 h p.i.	~2.3 (1 h p.i.)	N/A	[34]
[^99m^Tc]Tc-tricine-HYNIC/EDDA-SSS-GE11		Receptor binding and, internalization		Biodistribution at 1 and, 4 h p.i., gamma camera imaging at 1 h p.i.	3.6 ± 0.7 (1 h p.i.)	Good A549 tumor visualization at 1 h p.i.	[33]
[^99m^Tc]Tc-GGGC-GE11	A549	Cellular uptake, retention kinetics, internalization and blocking assays	Non-small-cell lung cancer (NSCLC)	Biodistribution at 1 and, 4 h p.i., SPECT scans at 1, 2, and 4 h p.i.	3.4 ± 0.5 (2 h p.i.)	The signal of A549 tumors reached its strongest at 2 h p.i.	[37]
[^99m^Tc]Tc-tricine-HYNIC-SSS-D4		Stability in solution and human serum, receptor binding, internalization assays		Biodistribution at 1 and, 4 h p.i., gamma camera imaging at 1 h p.i.	~7.6 (1 h p.i.)	Good A549 tumor visualization at 1 h p.i.	[78]
[^99m^Tc]Tc-tricine-HYNIC-D4		Stability, receptor binding		Biodistribution at 1 and, 4 h p.i.	~8.1 (1 h p.i.)	N/A	[77]
[^99m^Tc]Tc-tricine/EDDA-HYNIC-SSS-D4		Cellular uptake and blocking test		Biodistribution at 1 and, 4 h p.i.	~2.5 (1 h p.i.)	N/A	[76]
[^99m^Tc]Tc-SYPIPDT-ECG-TAMRA	NCI-H460	Receptor binding affinity, cellular uptake by microscopy	NSCLC	Gamma camera imaging and biodistribution at 1, 2, and 3 h p.i., fluorescent imaging and immunohistochemical staining	1.9 ± 0.1 (1 h p.i.)	2.7 ± 0.6 at 1 h p.i.	[83]
[^131^I]I-EEEEYFELV and [^131^I}I-DEDEYFELV	C6	Stability, partition coefficient, serum protein binding	Glioblastoma allografts	Biodistribution at 0.25, 1, and 2 h p.i.), binding, and internalization studies in brain homogenates	2.3 ± 0.2 ([^131^I]I-EEEEYFELV) and 0.6 ± 0.0 ([^131^I]I-DEDEYFELV) at 1 h p.i.	N/A	[50]

N/A: not available.

**Table 8:** Preclinical studies performed with PET tracers targeting integrin α_V_β_3._

Formulation	Cell Line	In Vitro Investigation	Murine Model	In Vivo Investigation	Biodistribution Tumor Uptake (%ID/g)	PET Imaging Tumor Uptake	Ref.
[^18^F]F-FPPRGD2	U-87 MG	Receptor-binding assay (IC_50_ = 51.8 ± 4.6 nM)	Glioblastoma	Biodistribution at 1 h p.i., and static microPET scans at 20 min, 1 and 2 h p.i.	2.3 ± 0.2	4.2 ± 0.2 (20 min)	[70]
[^18^F]F-Galacto-RGD		Receptor-binding assay (IC_50_ = 404 ± 38 nM)		Static microPET scans at 20 min, 1 and 2 h p.i.	N/A	2.1 ± 0.2 (20 min)	[70]
[^18^F]F-fluciclatide	EA-Hy926	Biding affinity (Ki = 2.3 nM)	Lewis lung Ccarcinoma (LLC)	Biodistribution and PET at 2 h p.i., blocking study	1.6 ± 0.1	1.5 ± 0.3	[92]
[^18^F]F-RGD-K5	U-87 MG	x	Glioblastoma	Metabolic stability, biodistribution, PET, blocking study	4.2 ± 0.6	1.4 ± 0.2 (U-87 MG); 1.2 ± 0.4 (A549) (SUV_max_)	[99]
[^18^F]F-RGD2		Receptor-binding assay IC_50_ = 2.3 ± 0.7 nM		Biodistribution at 0.5, 1 h, 2 h, and 4 h p.i., PET 1 h p.i.	4.3 ± 1.0 (2 h p.i.)	4.4 ± 0.6 (1 h p.i.)	[68]
[^18^F]F-RGD		Receptor-binding assay IC_50_ = 3.5 ± 0.3 nM		Biodistribution at 0.5, 1 h, 2 h, and 4 h p.i., PET 1 h p.i.	1.6 ± 0.4 (2 h p.i.)		[68]
[^18^F]F-Alfatide I		Receptor-binding assay IC_50_ = 46 ± 4.4 nM		Serum stability, biodistribution at 2 h p.i. and dynamic (2 -35 min) and static PET 1 and 2 h p.i., blocking study	2.3 ± 0.9 (2 h p.i.)	5.3 ± 1.2% (peak at 3 min p.i); 0.3 ± 0.1 (60 min p.i.)	[97]
[^18^F]F-Alfatide II	U-87 MG and MDA-MB-435	N/A	Glioblastoma or breast cancer	Dual PET imaging (^18^F-alfatide and ^18^F-FDG) of mice treated with doxorubicin or paclitaxel, computational modeling (ROI, time–activity curves, dual-tracer input function and tumor time–activity curve separation and kinetics.	N/A	4.7 ± 1.0%ID/g at 40 min	[98]
[^68^Ga]Ga-RGD	U-87 MG and A549	N/A	Glioblastoma and NSCLC	Biodistribution at 80 min p.i. and statistic PET at 1 h p.i.	2.9 ± 0.8 (U-87 MG) and 3.9 ± 1.2 (A549)	0.9 ± 0.3 (U-87 MG); 0.9 ± 0.3 (A549) (SUV_max_)	[99]
[^68^Ga]Ga-RGD2	U-87 MG and H727	N/A	Glioblastoma and neuroendocrine tumors	PET/CT scans, blocking and biodistribution (in major organs) at 1, 2 and 4 h p.i., dosimetry	N/A	2.2 ± 0.1 (U-87 MG); 1.5 ± 0.1 (A549) 1 h p.i.	[87]
	B16-F10	In vitro stability	Melanoma	Biodistribution at 10 min, 0.5 and 1 h p.i. and blocking studies at 0.5 h and clinical study	4.1 ± 0.9	N/A	[101]
[^68^Ga]Ga-3PRGD2	U-87 MG and LCC	N/A	LLC	PET (1 h p.i.), immunofluorescence, western blot	N/A	4.9 ± 1.2	[102]
[^68^Ga]Ga-TRAP-(RGD)_3_	MDA-MB-231	N/A	Breast cancer	Immunohistochemistry analysis, PET/CT at 1 h p.i., autoradiography and immunofluorescence imaging	N/A	3.0 ± 0.9	[103]
[^68^Ga]Ga-NOTA-PRGD2	U-87 MG	Receptor-binding assay (IC_50_ = 88.8 ± 5.4 nM)	Glioblastoma	Biodistribution at 1 h p.i. and PET imaging at 0.5, 1 and 2 h p.i.	7.0 ± 1.1	9.0 ± 2.0 (30 min)	[104]
[^68^Ga]Ga-NOTA-E[G_3_c(RGDfK)]_2_		Receptor-binding assay (IC_50_ = 61.6 ± 3.3 nM)		Biodistribution at 1 h p.i. and PET imaging at 0.5, 1, and 2 h p.i.	8.0 ± 0.9	10.1 ± 1.8 (0.5 h)	[104]
[^68^Ga]Ga-NOTA-RGD	SNUC4	Binding assay with human serum (Ki = 1.9 nM)	Colon cancer	Biodistribution 1 h p.i., blocking study and PET imaging at 1 and 2 h p.i.	1.6 ± 0.2	5.1 ± 1.0	[142]
[^68^Ga]Ga FSC-(RGD)_3_	M21	Stability, protein binding, binding affinity, internalization	Melanoma	Immunohistochemistry and PET/CT at 1 h p.i.	N/A	3.0 ± 0.9	[105]
[^68^Ga]Ga-HP_3_-(RGD)_3_		Binding assay (IC_50_ = 73 ± 22 nM)		Biodistribution 1 and 2 h p.i., blocking, and dynamic PET imaging for 1.5 h p.i., stability	6.1 ± 0.6 (1 h)	qualitative	[143]
[^64^Cu]Cu-RGD2	U-87 MG and H727	N/A	Glioblastoma and neuroendocrine tumors	PET/CT scans, blocking and biodistribution in major organs 1, 4, and 18 h p.i., dosimetry	N/A	2.3 ± 0.2 (U-87 MG); 1.5 ± 0.1 (H727) 1 h p.i.	[87]
[^64^Cu]Cu-DOTA- E[c(RGDfK)]_2_	U-87 MG	Binding assay (IC_50_ = 73 ± 22 nM)	Glioblastoma	Biodistribution 1 h p.i., PET imaging at 15, 30 min, 1, 2, 4, and 18 h p.i., dosimetry	9.9 ± 1.1 (0.5 h)	8.7 ± 1.5 (1 h p.i.)	[84]
[^64^Cu]Cu-CB-TE_2_A-c(RGDyK)	M21	Binding assay (IC_50_ = 6.0 ± 3.6 nM)	Melanoma	Biodistribution, blocking and PET imaging at 1, 2, 4 and 24 h pi	3.0 ± 0.9 (1 h p.i.)	qualitative	[75]
[^64^Cu]Cu-diamSar-c(RGDfD)		Binding assay (IC_50_ = 4.8 ± 0.9 nM)		Biodistribution, blocking and PET imaging at 1, 2, 4 and 24 h pi	1.5 ± 0.5 (1 h p.i.)	qualitative	
[^64^Cu]Cu-DOTA-PEG_4_-E[PEG_4_-c(RGDfK)]_2_	U-87 MG	Binding assay (IC_50_ = 74 ± 3 nM)	Glioblastoma	Biodistribution at 0.5, 1 and 2 h p.i., blocking and PET imaging at 1 h p.i.	8.2 ± 2.0 (0.5 h p.i.)	qualitative	[85]
[^64^Cu]Cu-DOTA-G_3_-E[G_3_-c(RGDfK)]_2_		Binding assay (IC_50_ = 62 ± 6 nM)		Biodistribution at 0.5, 1 and 2 h p.i., blocking and PET imaging at 1 h pi	8.5 ± 1.4 (0.5 h p.i.)	qualitative	
[^64^Cu]Cu-AmBaSar-RGD2		Binding assay (IC_50_ = 10.0 ± 0.5 nM)		PET imaging at 1, 2, 4, and, 20 h p.i., blocking and biodistribution at 20 h	1.8 ± 0.4	~2.5 (20 h)	[88]
[^64^Cu]Cu-NOTA-PEG_4_-SAA_4_-c(RGDfK)		Binding assay (IC_50_ = 444 ± 41 nM)		PET imaging at 0.5, and 2 and 4 h p.i, blocking and biodistribution at 4 h p.i	2.5 ± 0.2 (0.5 h p.i.)	1.1 ± 0.3	[52]
[^64^Cu]Cu-NOTA-(PEG)_2_-c(RGDfK)		Binding assay (IC_50_ = 288 ± 66 nM)		PET imaging at 0.5, and 2 and 4 h p.i, blocking and biodistribution at 4 p.i.	~4 (0.5 h p.i.)	2.4 ± 0.3	
[^125^I]I-bcRGD	U-87 MG and A549	Selective (2.1 ± 0.6) and binding assay (1.6% and 0.3% dose/mg for U-87 MG and A549)		Biodistribution at 10 min, 0.5, 1 and 2 h p.i.	3.8 ± 0.4 (U-87 MG); 2.1 ± 0.6 (A549) (0.5 h p.i.)	N/A	[106]

N/A: not available.

**Table 9:** Preclinical studies performed with SPECT tracers targeting integrin α_V_β_3._

Formulation	Cell Line	In Vitro Investigation	Murine Model	In Vivo Investigation	Biodistribution Tumor Uptake (%ID/g)	Ref.
[^99m^Tc]Tc-HYNIC-3G-RGD2	U-87 MG	Partition coefficient, binding assay U-87 MG (IC_50_ = 61.1 ± 2.1)	Glioblastoma and breast cancer	Biodistribution at 0.5, 1 and 2 h p.i., blocking, SPECT/CT and, metabolism	9.1 ± 1.8 (MDA-MB-435); 7.7 ± 1.2 (U-87 MG) (2 h p.i.)	[107]
[^99m^Tc]Tc-3PRGD2		Binding assay (IC_50_ = 2.4 ± 0.7 nM)	Glioblastoma	Biodistribution at 0.5, 1 and 2 h p.i., blocking, SPECT/CT (0.5, 1, 2 and 4 h p.i.) and, metabolism	9.7 ± 3.2 (2 h p.i.)	[110]
[^99m^Tc]Tc (RGD_echi_-Cys)(PNP)_43_		Flow cytometry, cell uptake		Biodistribution at 0.5 and 2 h p.i. and metabolism	~0.4 (2 h p.i.)	[112]
[^99m^Tc]Tc-HYNIC-RGD	M21	Protein binding in fresh human plasma, binding affinity	Melanoma	Biodistribution at 1 and 4 p.i. and planar γ-camera (10 min) imaging	2.1 ± 0.4 (M21); 1.5 ± 0.3 (A549) (4 h p.i.)	[113]
[^99m^Tc]Tc-RAFT-RGD	B16F0- and TS/A-pc	Immunoprecipitation, western blot, blood distribution	Melanoma and mammary cancer	Biodistribution, immunohistochemistry, autoradiography, planar γ-camera and SPECT imaging	2.4 ± 0.5 (B16F0); 2.7 ± 0.8 (TS/A-pc) (1 h p.i.)	[115]
[^99m^Tc]Tc-RAFT-RGD	DAOY-Luc spheroids or HD-MB03-Luc	Experiments involving the generation of radioresistant medulloblastoma cell lines	Intracranial orthotopic	SPECT and bioluminescence	N/A	[114]
[^99m^Tc]Tc (HYNIC-E E[c(RGDfK) _2_] _2_)(tricine)(TPPTS)]	MDA-MB-435	Solution stability, partition coefficient, binding assay (IC_50_ = 51 ± 11 nM)	Breast cancer	Biodistribution at 5 min, 0.5, 1 and 2 h p.i., SPECT at 1, 2 and 4 h p.i. and metabolism	7.3 ± 1.3 (2 h p.i.)	[116]
[^99m^Tc]TcO(MAG_2_-3G_3_-E{c(RGDFk)_2_	U-87 MG	Binding assay (IC_50_ = 3.6 ± 0.6 nM), partition coefficient	Glioblastoma	Biodistribution at 0.5 and 2 h p.i., planar imaging and metabolic stability	8.3 ± 1.5 (2 h p.i.)	[117]
[^99m^Tc]Tc-Galacto-RGD2		Binding assay (IC_50_ = 20 ± 2 nM)		Biodistribution at 5, 30, 60 and 120 min p.i., blocking, SPECT/CT at 1 h p.i. and metabolism, immunostaining, immunohistochemistry	5.6 ± 1.5 (2 h p.i.)	[118]
[^111^In]In-RGD_2_/[^111^In]In-DOTA-E-[c(RGDfK)]_2_	FaDu, SCCN_ij3_, and SCCN_ij20_	N/A	HNSCC	Biodistribution at 1 h p.i., immunohistochemistry, autoradiography and SPECT/CT (3 frames of 20 min)	2.2 ± 0.0 (FaDu), 1.9 ± 0.5 (SCCN_ij3_), and 1.2 ± 0.0 (SCCN_ij20_) (1 h p.i.)	[120]
[^111^In]In (DOTA-Galacto-RGD2)	U-87 MG and MDA-MB-435	Binding assay U-87 MG (27 ± 2 nM)	Breast cancer	Biodistribution at 1, 4, 24 and 72 h, planar imaging at 1, 4 and 24 h p.i., blocking, immunostaining, immunohistochemistry	6.8 ± 1.0 (1 h p.i.)	[122]
[^111^In]In-3P-RGD2		Binding assay U-87 MG (29 ± 4 nM)		Biodistribution at 1, 4, 24 and 72 h, planar imaging at 1, 4 and 24 h p.i., blocking, immunostaining, immunohistochemistry	6.2 ± 1.6 (1 h p.i.)	[122]
[^111^In]In-DOTA-RGD2	NT2.5 and MDA-MB-231	Serum stability		Biodistribution at 0.5, 1.5, 3, 6 and 24 h	4.8 ± 0.7(NT2.5), 2.2 ± 0.9 (MDA-MB-231) (3 h p.i.)	[124]

N/A: not available.

**Table 10:** Preclinical studies performed with radiotracers targeting integrin α_V_β_3_ aiming therapy.

Formulation	Therapy Dose (MBq)	Murine Model	In Vivo Investigation	Main Findings	Ref.
[^177^Lu]Lu-DOTA-E-c(RGDfK)	37	OVCAR-3 ovarian cancer	Survival study	Treated mice survived 16 weeks more than the untreated group.	[125]
[^177^Lu]Lu-NOTA-SCN-c(RGDyK)	0.37	CT-26 colon cancer	Biodistribution (at 1, 2, 4, 12 and 24 h p.i.)	The tumor uptake and the tumor/muscle ratio at 1 h p.i. was 1.7 ± 0.3 and 2.1 ± 0.4%ID/g. The highest uptake was observed in kidneys 7.6 ± 0.7%ID/g.	[126]
[^177^Lu]Lu-3PRGD_2_	0.37, 37.74 and 111	LCC tumor model	Biodistribution (at 1, 4, 24 and 72 h p.i.), gamma imaging (at 4 and 24 h p.i.) and maximum tolerated dose (MTD), immunohistochemistry and hematoxylin-eosin staining	The tumor uptake at 1 h p.i. was 6.0 ± 0.6%ID/g and remained at 1.2 ± 0.2%ID/g 72 h p.i. Highest uptake was observed in the intestine (5.2 ± 0.5%ID/g) and kidney (4.2 ± 1.1) at 1 h p.i. The MTD was greater than 111 MBq per mouse.	[127]
[^90^Y]Y-RAFT-RGD	30–37 (1× or fractionated 2×)	U-87 MG glioblastoma	Biodistribution at 1, 4, 24 and 48 h p.i. of 0.37 MBq of [^90^Y]Y-RAFT-RGD, toxicity and, dosimetry	The tumor uptake of [^90^Y]Y-RAFT-RGD 1 h p.i. was quick and high (9.0 ± 4.3% ID/g) and remained at 1.8 ± 0.7% ID/g 48 h p.i. The highest kidney uptake was 13.9 ± 3.5%ID/g at 1 h p.i. The toxicity findings were as follows: reduction in leukocyte and platelet counts and higher serum creatinine levels in the treated groups (compared to control). Radiation dosimetry extrapolation to humans: the whole-body effective dose was estimated at 0.11 mSv/MBq.	[128]
[^177^Lu]Lu-RAFT-RGD	30–37		SPECT/CT (at 1 and 4 h p.i.), toxicity	The tumor uptake at 1 and 4 h p.i. was 3.3 ± 0.5%ID/g and 3.8 ± 0.9%ID/g, and remained at 1.6 ± 0.0%ID/g 48 h p.i. The tumor/muscle ratio was ~10 at 1 h p.i.The highest activity levels (~6%ID/g) were detected in the kidneys and the bladder. Toxicity findings: reduction in leukocyte and platelet counts in treated groups (compared to control).	
[^177^Lu]Lu-5p-C-NETA-c(RGDyK)	2.22		Biodistribution at 1, 4 and, 24 h p.i	Tumor uptake was 1.4 ± 0.6% ID/g 1 h p.i. The kidneys presented the highest uptake 1 h p.i. (3.2 ± 0.5%ID/g)	[129]
[^90^Y]Y-NMEB-RGD	7.4, 3.7 and 1.75 MBq		Antitumoral radiotherapy efficacy, TUNEL and hematoxilin-eosin staining	The treated group presented lower tumor volumes. The tumor vasculature was lower in the group receiving a medium dose of ^90^Y-NMEB-RGD. Also, this group presented more cell apoptosis.	[130]
[^177^Lu]Lu-RGD	18.5 and 29.6	Patient-derived xenografts (PDX) from NSCLC	SPECT imaging (at 4, 24, 48, 72, and 96 h p.i.) and biodistribution (at 4, 24, 48 and, 72 h p.i.), therapy regimen, CD31 immunohistochemistry	[^177^Lu]Lu-EB-RGD demonstrated higher tumor uptake than [^177^Lu]Lu-RGD (13.4 ± 1.0 vs. 2.6 ± 1.6%ID/g). The [^177^Lu]Lu-EB-RGD group showed more cell apoptosis than control and [^177^Lu]Lu-RGD. A therapy dose of 18.5 MBq [^177^Lu]Lu-EB-RGD might be strong enough.	[131]
[^177^Lu]Lu-EB-RGD					
[^225^Ac]Ac-DOTA-cRGDfK	0.037	HER2 (NT2.5 and MDA-MB-231) breast cancer	The estimation of maximum tolerated activity (eMTA), biodistribution in normal tissues, α-Camera	The organs receiving the highest mean absorbed dose were kidneys (2.5 Gy) > spleen (1.8 Gy) > liver (1.2 Gy). The eMTA was 150 kBq (kidneys as the limiting tissue). The decay of daughters (^213^Bi and ^221^Fr) was also monitored. All the free ^221^Fr and the majority of the ^213^Bi decayed at 3 h.	[124]

**Table 11:** Clinical trials using radiolabeled RGD-based peptides.

Agent	Clinical Trials	SUV_max_	Cancer Type	No. of Patients	Clinical Phase/Study Period	Radio-Dose	Toxicity	Imaging Tool	Comments	Ref.
[^99m^Tc]Tc-RGD-SCRGDSY/[^99m^Tc]Tc-αP2)	N/A	N/A	Metastatic melanoma	14 (10 men and 4 women)	Up to 12 months	185 to 1222 MBq	No adverse effect	SPECT/CT	Tumor detection.	[146]
[^18^F]F-Galacto-RGD	N/A	Ranged in tumors (1.2 to 9.0)	Malignant melanoma, sarcomas, and osseous metastases	19 (9 men, 10 women)	Phase I/II	133–200 MBq	N/A	PET	Demonstrate a highly favorable biodistribution in humans with specific receptor binding, and visualization of α_v_β_3_ expression in tumors with high contrast.	[147]
[^99m^Tc]Tc-3 PRGD2	N/A	N/A	Differentiated thyroid cancer patients with radioactive iodine refractory lesions (RAIR-DTC)	10 (2 men and 8 women)	N/A	11.1 MBq/kg	N/A	SPECT/CT	[^99m^Tc]Tc-3PRGD2 with SPECT is a promising modality for diagnosing and guiding further treatment of RAIR DTC.	[148]
[^18^F]F-FPPRGD2	NCT01806675	0.8–5.8	Glioblastoma multiforme recurrence	17 (8 men and 9 women)	Phase I/II	The [^18^F]F-FPPRGD2 doses at injection ranged from 3.8 to 9.9 mCi (mean, 8.1 mCi ± 1.7)	Safe	PET/CT	Function as increase uptake of glioblastoma.	[149]
[^18^F]F-alfatide	N/A	5.4 ± 2.2	Lung cancer	26	N/A	(213.3 ± 29.8 MBq)	Safe	PET/CT	Effective in the diagnosis of NSCL.	[150]
[^68^Ga]Ga-NODAGA-RGD	N/A	N/A	Hepatocellular carcinoma	9 men	Phase I	154–184 MBq	Well tolerated and metabolically stable in humans	PET/CT	Uptake in HCC tumors was not enough.	[151]
[^68^Ga]Ga-NODAGA-RGD2	N/A	>10	Locally advanced breast cancer	5 women (33 to 68 years old)	N/A	111–185 MBq	N/A	PET/CT	Good uptake in tumor site.	[101]
	NCT02970786	4.5–17.7	Neuroendocrine neoplasms and breast cancer	10 (5 with neoplasm and 5 with breast cancer)	Phase I	97.3–220 MBq	Safe.	PET/CT	Good image contrast and stable retention in tumor	[152]
	NCT03271281	1.4–14.1	Neuroendocrine neoplasms	113	Phase II	104–226 MBq	No grade 3–5 adverse events	PET/CT	Great tumor uptake	[153]
[^18^F]F-RGD-K5	NCT01447134	For main tumor (5.3–6.0) For nodal (3.3–4.7)	Head and neck carcinoma (HNC)	11	Phase II	10 mCi	N/A	PET/CT	Assessing response to concurrent chemoradiotherapy (CCRT) in patients with advanced HNC.	[154]
[^18^F]F-FPPRGD2	NA	3.7 ± 1.3	Cervical and ovarian tumors	6 women	NA	Ranged from 196 to 344 MBq	N/A	PET/CT	Have potential for early prediction of response to treatment.	[89]
[^18^F]F-ALF-NOTA-PRGD2	NCT03384511	5.4 ± 2.6	Lung cancer, stomach cancer, cervical cancer, gall bladder cancer, breast cancer, nasopharyngeal carcinoma, soft tissue carcinoma, esophageal cancer	38 (only 25 met criteria)	Phase IV	224.6 ± 38.2 MBq	N/A	PET/CT	Predictive for treatment response of antiangiogenic treatment (apatinib).	[155]
[^99m^Tc]Tc-3PRGD2	NCT02744729	Malignant vs. benign lesions (5.1 ± 2.0 vs. 2.0 ± 0.7)	Esophageal cancer	29 (24 men and 5 women)	Early phase I	11.1 MBq/kg	N/A	SPECT/CT	Valuable for the diagnosis and staging of esophageal cancer.	[156]
[^68^Ga]Ga-RGD	NCT04222543	Ranged between 4.0 and 12.7	Oral squamous cell carcinoma (OSCC).	10	Phase II	214 ± 9 MBq	N/A	PET/CT	Provide insight in angiogenesis as a hallmark of the head and neck squamous cell carcinomas’ tumor microenvironment.	[157]
[^68^Ga]Ga-NOTA-3P-TATE-RGD	NCT02817945	27.2 ± 13.6	Gastroenteropancreatic–neuroendocrine tumors (GEP-NETs)	35	Early phase I	74–148 MBq	N/A	PET/CT	Detection of liver metastases.	[158]
[^68^Ga]Ga-FAPI-RGD	NCT05543317	Primary tumors: SUV_max_ 18.0 and lymph node metastases: SUV_max_ 12.1	Nasopharyngeal carcinoma, small lung cancer, pancreatic cancer; lymph node, brain, lung, liver, bone, and subcutaneous metastasis.	22	NA	3.0–3.7 MBq/kg	Safe and well tolerated	PET/CT	Imaging of various cancer types and functions to increase tumor uptake.	[159]

N/A: not available.
